# The impact of energy poverty on the health and welfare of the middle-aged and older adults

**DOI:** 10.3389/fpubh.2024.1404014

**Published:** 2024-08-16

**Authors:** Yibo Wang, You Wu, Chenyu Wang, Li Li, Yalin Lei, Sanmang Wu, Zhi Qu

**Affiliations:** ^1^School of Economics and Management, China University of Geosciences, Beijing, China; ^2^Key Laboratory of Carrying Capacity Assessment for Resource and Environment, Ministry of Natural Resources of the People’s Republic of China, Beijing, China

**Keywords:** energy poverty, health, welfare, OLS regression, policies

## Abstract

Drawing upon data from the 2018 CHARLS, this paper utilizes MEPI and a 10% threshold indicator to, respectively, assess the energy poverty (EP) status among middle-aged and older adults in China, focusing on the unavailability and unaffordability of energy services. Additionally, an econometric model is constructed to investigate the effects of EP on the health and welfare of middle-aged and older adults. Regression results indicate that EP exerts a significant negative impact on the health and welfare of middle-aged and older adults. This conclusion remains robust after conducting endogeneity and robustness tests, demonstrating its validity. Finally, based on the calculation results, we propose relevant policy recommendations including enhancing energy services for older adults in rural areas, integrating household energy alternatives with targeted poverty alleviation, enhancing monitoring mechanisms, and conducting energy education activities to alleviate EP and improve the quality of life of middle-aged and older adults.

## 1 Introduction

Eliminating all forms of poverty globally is one of the United Nations Sustainable Development Goals, and EP, as a typical manifestation of poverty, has long been a hot topic of concern for scholars. According to the World Bank’s 2018 report, nearly 3 billion people worldwide still lean on polluting fuels to meet their energy needs, indicating a severe international situation regarding EP. In China, energy demand occupies a central position in people’s daily lives, serving as both a basic necessity for a better life and a necessary material foundation for achieving the goal of common prosperity. According to the International Energy Agency’s 2020 report, although China achieved 100% electricity coverage in 2020, only 71.3% of residents use clean cooking facilities. Issues such as low energy efficiency, poor energy structure, and weak energy capacity still exist. In January 2020, the State Council’s executive meeting emphasized the urgency of studying and establishing long-term mechanisms to address relative poverty issues, consolidating poverty alleviation achievements. Inequality in energy consumption can lead to health disparities, affecting the work capacity of energy-poor groups and further impacting social equity to a large extent.

The issue of EP in China has long been a subject of extensive attention from both domestic and international scholars. Research in this area has been concentrated on three main aspects: analysis of the current status of EP, factors influencing household energy choices, and assessment of health risks associated with solid fuel usage. Regarding the current status of EP, a Chinese regional EP assessment system has been developed based on accessibility, cleanliness, management completeness, affordability, and efficiency (1). Analyses of cooking energy usage in rural Chinese households using census data were conducted (2), alongside questionnaire surveys on household energy usage in Pan County, Guizhou Province, Nujiang Prefecture, Yunnan Province, and 26 provinces nationwide (3–5). These studies indicate that rural Chinese households rely predominantly on non-clean fuels for cooking and heating. Studies have revealed shifts from coal to electricity, liquefied gas, and natural gas, and from firewood to electricity and liquefied gas, but a significant proportion of households continued to use solid fuels (coal and firewood) for cooking in 2011 (6).

Residents experiencing EP often rely on traditional biomass, coal, and other solid fuels for cooking and heating purposes. However, the combustion of these fuels releases substantial amounts of harmful gases, leading to indoor and outdoor air pollution and posing severe health risks to residents’ respiratory systems. Additionally, those living in EP face the burden of allocating more time and income to sustain basic household energy consumption. Consequently, this situation exacerbates disparities in their quality of life, affecting aspects such as leisure activities, social interactions, and residential environments, ultimately contributing to a lower overall welfare level. Especially concerning the middle-aged and senior citizens, they are more vulnerable to energy poverty-related issues. However, existing literature on the impact of energy poverty on this age group is very limited; most scholars’ research has focused on the general population. Furthermore, there is also a scarcity of studies in existing literature that explore the effects of energy poverty on residents’ welfare. Therefore, the novelty of this paper lies in selecting the middle-aged and older adults as the research subjects. Based on the CHARLS^1^ 2018 data, we simultaneously employ the MEPI index and a 10% threshold to assess the energy poverty status of this population. Additionally, we use econometric models to explore the concurrent effects of energy poverty on their health and welfare and ultimately puts forward pertinent policy recommendations.

## 2 Literature review

### 2.1 Research progress on the definition and measurement of EP

The issue of EP has long been a concern for experts and scholars, yet there remains no internationally unified definition. It is also articulated as fuel poverty, with its origins tracing back to the fuel rights movement in the UK during the 1970s. Fuel poverty is primarily characterized by the inability, due to economic constraints, to maintain warmth within the home (7). It is further defined as the incapacity to afford adequate warmth due to poor energy efficiency, wherein individuals classified as fuel-poor typically spend over 10% of their income to achieve the minimum acceptable indoor comfort levels (21°C for kitchen and bathroom areas, 16°C for bedrooms) (8). Furthermore, Boardman introduced the 10% indicator, suggesting that if a household needs to allocate more than 10% of its income to meet energy services, it is considered an energy-poor household. Additionally, fuel poverty is described as households being unable to meet their basic energy needs, often quantified by measuring energy usage levels below the poverty line based on expenditure or income (9). The overarching feature of fuel poverty is identified as the disproportionate cost of meeting essential energy needs compared to the social average, with remaining income often falling below the official economic poverty line (10).

The International Energy Agency (IEA) also contributed to the conceptualization of EP in 2002 (11), offering a more specific definition. According to the IEA, EP encompasses situations where individuals rely on traditional biomass energy sources like firewood, straw, rice husks, or other agricultural waste, as well as livestock manure for cooking or heating purposes. Additionally, it includes those who lack access to modern electricity services. This definition highlights the dual challenge faced by many individuals in terms of both the type of energy sources they use for daily activities and their access to modern, reliable electricity. The definition is revised by substituting reliance on traditional biomass energy with the consumption of traditional cooking equipment using biomass energy (12). Various methodologies have been embraced by researchers to assess energy deprivation, including the “10% Principle,” “LIHC Index,” MEPI, and Basic Needs Approach. While prevalent in developed nations, the “10% Principle” and “LIHC Index” tend to overly emphasize the influence of income on EP. The adoption of the Energy Deprivation Index (EDI) is advocated to evaluate EP in developing nations (13). The Multidimensional EP Index (MEPI) was devised comprising five dimensions and six indicators of fundamental energy services (14). Although the MEPI approach has been utilized by numerous scholars to gauge EP, challenges persist in accessing relevant microdata or indicators. A multidimensional EP index was formulated to evaluate EP in developed nations, taking into account factors like energy expenditures, income levels, and residential energy efficiency (15). Moreover, the Basic Needs Approach denotes the minimum energy consumption required to sustain daily household activities and holds the advantage of computing threshold values based on objective energy consumption data. Nevertheless, it fails to consider the reality that inhabitants in diverse regions exhibit varying energy consumption levels due to economic development, climate conditions, and other factors. Currently, a systematic approach is lacking to precisely delineate EP thresholds for distinct regions and demographics. Due to the vast geographical span of our country, there are significant differences in economic development, cultural lifestyles, and customs among various regions. Therefore, the measurement and resolution of energy poverty issues in China are particularly complex. Due to China’s large geographical span, different levels of economic development in various provinces and cities, and large differences in cultural and living customs, this paper adopts the MEPI index to more comprehensively respond to the EP status of China’s middle-aged and older adults, and with reference to the scholars’ method of calculating the EP status of Panxian County in Guizhou Province, this paper also adopts a 10% indicator to compare with the MEPI index to account for China’s middle-aged and older adults EP situation.

### 2.2 Research progress on the impact of EP on health and welfare

The literature on the health impacts of EP is vast. Firstly, studies, categorized by the nature of health, not only focus on the physiological effects of EP but also delve into its implications for mental health. Secondly, while most research has concentrated on the impact of EP on the health status of adults, in recent years, scholars have begun to investigate the causal relationship between EP and the health of children who are not yet fully developed and have poorer physical resistance. Thirdly, studies emphasizing gender differences in the impact of EP have focused on its effects on women’s health. Research by the World Health Organization also indicates that indoor air pollution is a primary cause of respiratory diseases and deaths among household members. The earliest investigations into the health effects of EP also originated from the field of public health, with a focus on the increased incidence and mortality rates of respiratory diseases among household members due to the scarcity of clean energy. In daily household activities, residents primarily use non-clean fuels such as crop residues, animal dung, firewood, and coal, and the harmful gases or particles released during combustion increase the incidence and mortality rates of respiratory diseases among household members (16–22). The use of non-clean energy sources has significant health implications due to the serious pollution caused by low household combustion efficiency and poor ventilation. Indoor air pollution is characterized by elevated levels of carbon monoxide, aromatic compounds, and suspended particles. PM10 particles can easily penetrate the respiratory system, causing harm to health. More concerning is that PM2.5 particles can deposit in the deepest parts of the respiratory system, leading to even more severe effects. In addition to traditional research focusing on the impact of EP on the respiratory system, recent studies have also found that using non-clean energy sources can harm other bodily systems. For example, recent research has focused on demonstrating that the use of non-clean energy sources can cause certain levels of cognitive impairment (23, 24). Many scholars have also discovered the detrimental effects of using non-clean energy sources on the cognitive function of Chinese residents (25–29). Additionally, literature has investigated the cardiovascular and cerebrovascular diseases caused by the use of non-clean energy sources (30, 31). In recent years, fields such as health economics and energy economics within applied economics have conducted extensive causal identification studies on the relationship between EP and residents’ physical health. For instance, Oum (32) investigated the causal relationship between household EP in Laos and the likelihood of family members suffering from acute or chronic diseases, finding that the inability to access electricity increases the probability of family members falling ill. Other scholars have examined the impact of EP on self-rated health and found that it significantly reduces the health status of household members (33–35) (36, 37). The research mentioned above has predominantly focused on developing countries, where EP mostly manifests as lack of access to energy sources. Additionally, there is literature primarily addressing the impact of EP on the health of residents in developed countries (38–41). In these countries, the characteristic feature of EP is affordability-based (fuel poverty), and the main mechanisms through which EP affects health are discussed from the perspectives of indoor warmth, sleep quality, etc. For example, in the UK, where winters are long and relatively cold, residents who cannot afford energy costs beyond their means experience indoor coldness, leading to a sharp increase in the risk of respiratory diseases, cardiovascular diseases, etc. (42). Furthermore, there have been some recent novel findings regarding the relationship between EP and health. For instance, Prakash and Munyanyi (43) analyzed the causal relationship between EP and obesity among Australian adults, finding that EP significantly increases the incidence of obesity in adults, mediated through mechanisms such as sleep duration, health status, and psychological stress. In addition to the impact on physical health, scholars have also focused on the influence of EP on mental health (44–46). For example, Zhang et al. (46) analyzed the relationship between multidimensional EP and residents’ health in China, discovering that compared to rural areas, EP has a more severe detrimental effect on the mental health of urban residents.

In studies examining the impact of EP on residents’ welfare, differences in the definition and understanding of welfare among various schools of thought lead to variations in the selection of indicators and proxy variables in empirical research. Consequently, based on different definitions of welfare, literature related to the impact of EP on residents’ welfare can be categorized into three parts: The first set of literature analyzes the impact of EP on objective welfare. Within the framework of objective welfare theory, residents’ welfare implies their disposable income or the share of resources they enjoy. Therefore, when energy prices rise and the share of energy resources they enjoy is low, residents’ welfare is also compromised. The second set explores the direct impact of EP on subjective wellbeing. This portion of the literature advocates for the use of happiness economics as a theoretical foundation and typically considers individuals’ subjective wellbeing as a proxy variable for personal welfare. The third set of literature, based on Sen’s capability theory, primarily investigates the impact of EP on specific capabilities or capacities of individuals, such as physical and mental health, children’s education, and employment opportunities.

## 3 Model methodology and data sources

### 3.1 Assessing EP

As discussed earlier, current research on EP can generally be divided into two directions: the inaccessibility of modern energy and the unaffordability of living fuels. This article considers both the inaccessibility and unaffordability of modern energy when measuring EP. After calculating the degree of inaccessibility and unaffordability of energy for each middle-aged and older adults, we obtain the level of EP at the provincial level by calculating the average. In the empirical analysis section, we use the inaccessibility of energy as the core explanatory variable in the baseline regression model. Meanwhile, in the robustness test, we adopt the method of replacing explanatory variables, using the unaffordability of energy as the core explanatory variable, to study the impact of EP from another angle, ensuring the robustness of the results.

#### 3.1.1 Inaccessibility

This article follows the approach of Alkire and Foster (47) and Nussbaumer et al. (14), using MEPI to assess the inaccessibility of modern energy services for middle-aged and older adults. Taking into account China’s actual conditions, five dimensions and nine indicators representing basic energy services were selected to compose the index. Each dimension is equally weighted at 0.2. The specific details are as follows in Table 1.

**Table 1:** Multidimensional EP index system and related explanations.

Dimension	Indicator (weight)	Indicator meaning	Deprivation threshold explanation
Cooking	Cooking fuel (0.2)	Types of fuels used for cooking	Using any fuel other than electricity, liquefied petroleum gas (LPG), natural gas, town gas, or biogas
Lighting	Electricity (0.2)	Availability of electricity	No
Entertainment/Education	Television (0.2)	Ownership of a television	No
Housing	Refrigerator (0.05)	Ownership of a refrigerator	No
	Water heater (0.05)	Ownership of a water heater	No
	Air conditioner (0.05)	Ownership of an air conditioner	No
	Washing machine (0.05)	Ownership of a washing machine	No
Communication	Mobile phone (0.1)	Ownership of a mobile phone	No
	Broadband (0.1)	Access to broadband	No

The specific steps to construct the MEPI mathematical model are as follows:

1. Assuming there are n individuals in the older adults and d variables, let $Y=\left[y_{ij}\right]$ represent the n×d dimensional achievement matrix depicting individuals across different variables. Here, each row vector $y_{i}=\left(y_{i1},y_{i2},\cdots ,y_{\mathrm{id}}\right)$ represents individual achievements on different variables, and each column vector $y_{j}={\left(y_{1j},y_{2j},\cdots ,y_{nj}\right)}^{T}$ represents the achievement distribution of a given variable across different individuals. The weight vector w is composed of elements $w_{j}$ corresponding to weights attached to different variables, and $\overset{d}{\underset{j=1}{\sum }}w_{j}=1$ .
2. We define $z_{j}$ as the deprivation threshold for variable j . In this context, the deprivation threshold comprises a series of conditions that need to be satisfied (refer to Table 1). When the conditions in the table are met, variable j for individual i will be considered deprived. Let $g=\left[g_{ij}\right]$ be the deprivation matrix, where the elements of the matrix take the following values:

(1) $$g_{ij}=\{\begin{array}{c} w_{j},\mathrm{Variable}\;j\;\mathrm{on}\;\mathrm{individual}\;i\;\mathrm{is}\;\mathrm{deprived}\\ 0,\mathrm{Variable}\;j\;\mathrm{on}\;\mathrm{individual}\;i\;\mathrm{is}\;\mathrm{not}\;\mathrm{deprived} \end{array}$$

3. Construct the column vector $c_{i}$ to represent the weighted sum of deprivation experienced by individual i , i.e., $c_{i}=\overset{d}{\underset{j=1}{\sum }}g_{ij}$ . Then, define a threshold value k . If the weighted deprivation index $c_{i}$ exceeds k for individual i , that person is considered to be in EP. Therefore, the value of $c_{i}\left(k\right)$ is:

(2) $$c_{i}\left(k\right)=\{\begin{array}{c} c_{i},c_{i}>k\\ 0,c_{i}\leq k \end{array}$$

4. Calculate the incidence rate of EP H in the population using the following method:

(3) $$H=\frac{q}{n}$$

Where q represents the number of individuals in EP, and n is the total population.

Calculate the EP intensity A using the following formula:


(4) $$A=\overset{n}{\underset{i=1}{\sum }}c_{i}\left(k\right)/q$$

Finally, the MEPI index can be calculated according to Equations (1) to (4), that is, MEPI=H×A .

In this study, we refer to the work of Nussbaumer et al. (14) and set the threshold value k as 0.3. Since the MEPI index is at the macro level, we can only use the MEPI index to calculate the EP intensity at the provincial level. In the subsequent empirical analysis, to measure the unavailability of modern energy for individual older adults, we use the weighted deprivation index $c_{i}$ and introduce two variables: the dummy variable access01, which takes the value of 1 if the weighted deprivation index $c_{i}$ exceeds k , and 0 otherwise; the continuous variable access02, which equals the value of the weighted deprivation index $c_{i}$ for individual i . A higher value indicates a more severe situation of energy unavailability in the household.

#### 3.1.2 Energy unaffordability

This paper utilizes Boardman (8) 10% indicator as a metric to gauge the affordability of modern energy among the older adults. As indicated by Lin et al. (44) study, the 10% indicator proves to be an effective tool for evaluating EP in the context of China. This indicator assesses whether a household faces EP by analyzing the ratio of household energy expenditure to income. Typically, a threshold of 10% is established, and if the proportion of energy expenditure to income surpasses this threshold, the household is classified as experiencing EP.

We also establish two variables to gauge the affordability of energy: The dummy variable afford01 takes the value of 1 if the ratio of energy income exceeds a specific threshold (i.e., 10%), and 0 otherwise; The continuous variable afford02, formed by dividing energy expenditure by total income, is used to reflect the depth of poverty. A higher ratio indicates a more severe situation of energy unaffordability in the household.

#### 3.1.3 Results of EP measurement

**Figure 1:**
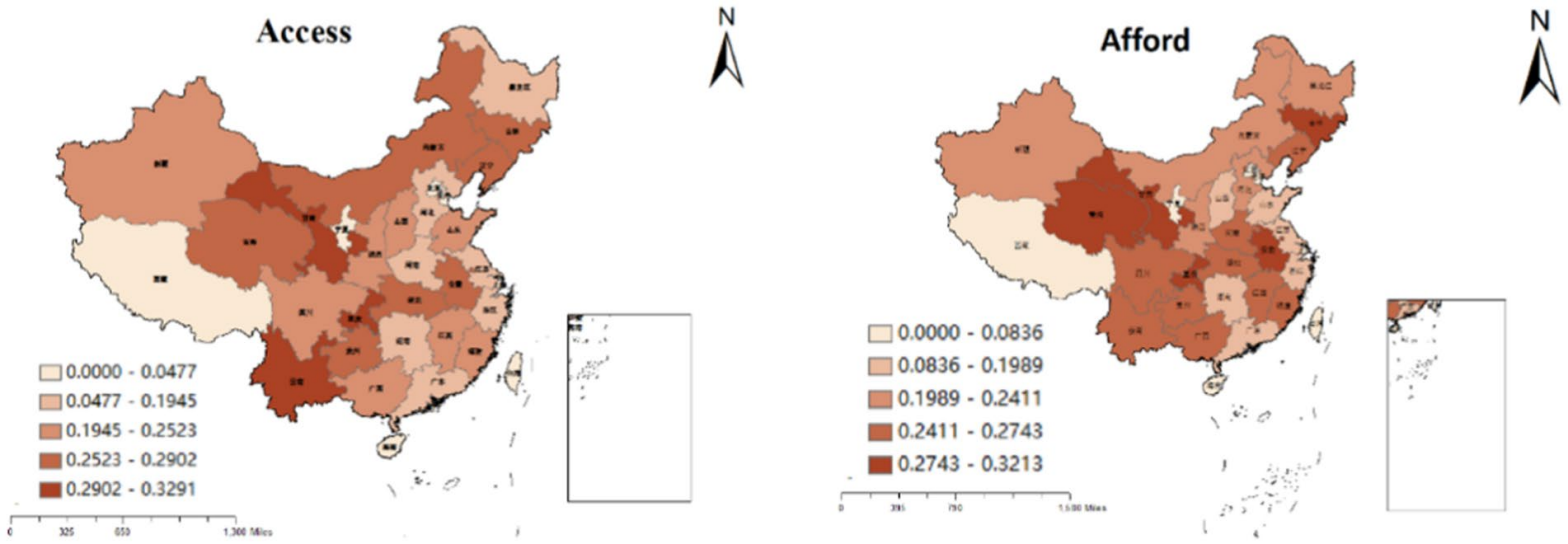
EP intensity across provinces in China.

Using the aforementioned measurement approaches, we assessed the EP among respondents in the China Health and Pension Tracking Survey. Furthermore, we calculated the provincial EP intensity across 28 provinces (data for Tibet, Ningxia, Hainan, Taiwan, and other provinces are not available) by computing the average values. The specific results are depicted in Figure 1.

The figure illustrates a consistent EP intensity measured by both methods across all provinces in China. Moreover, it highlights a notable disparity, with the EP intensity in the eastern coastal areas being significantly lower compared to that in the western regions.

### 3.2 Data source

The data utilized in this paper is derived from the 2018 edition of the China Health and Retirement Longitudinal Study (CHARLS). This research initiative was launched in 2011, spanning 28 provinces, 150 county-level units, and 450 village-level units across the nation. It encompassed around 10,000 households and 17,000 individuals, aiming to assemble a collection of high-quality microdata representing middle-aged and older adults aged 45 and above in China. The survey encompasses various aspects, including respondents’ fundamental demographics, health status and functionality, property and housing conditions, as well as household income and expenditure. The section dedicated to health status and functionality provides insights into respondents’ health and welfare levels, while the unit focusing on household income and expenditure compiles data on energy consumption. This compilation of data is essential for investigating the ramifications of EP on the health and welfare of the middle-aged and older adults.

In line with the requirements of this study, the data was organized and invalid samples were eliminated, ultimately selecting 6,748 valid samples for analysis.

### 3.3 Variable definitions and descriptive statistics

In this study, residents’ self-rated health status serves as a proxy for their health, while residents’ life satisfaction represents their overall welfare.

To investigate the influence of EP on the health and welfare of middle-aged and older adults, we selected the inaccessibility and unaffordability of modern energy as explanatory variables. Drawing on existing literature that links EP to residents’ welfare, using subjective wellbeing as an explanatory variable (48), this paper employs the theory of happiness economics to consider residents’ life satisfaction as a proxy variable for individual welfare. Additionally, we controlled for individual characteristics of respondents, family assets (ownership of a house, housing area, etc.), social connections (family size, etc.), and other relevant factors. The definitions and statistical properties of each variable are presented in Table 2.

**Table 2:** Variable definitions and descriptive statistics.

Index	Variable	Label	Mean	Standard deviation	Minimum	Maximum
Wellbeing	Life-satisfaction	Welfare	2.73	0.786	1	5
Health	Self-rated health	Health	2.942	1.022	1	5
Energy poverty	Inaccessibility	Access01	0.226	0.187	0	1
		Access02	0.386	0.487	0	1
	Unaffordability	Afford01	0.276	0.326		
		Afford02	0.443	0.497	0	1
Individual characteristics	Gender (1 = Male, 0 = Female)	Gender	0.534	0.499	0	1
	Age	Age	63.034	8.909	46	108
	Education level	Education	1.805	1.398	0	6
	Marital status (1 = Married, 0 = Unmarried)	Marital_s	0.872	0.334	0	1
	Urban residence (1 = Yes, 0 = No)	City	0.132	0.338	0	1
	Northern region (1 = Yes, 0 = No)	North	0.599	0.49	0	1
Economic status	Housing	House	0.767	0.423	0	1
	Housing area	House_s	272.277	244.19	0	2,280
Social relations	Family Size	Number	3.152	1.545	1	13

## 4 Results and discussion

### 4.1 Model specification

Self-rated health level and life satisfaction were treated as continuous variables, and the following Ordinary Least Squares (OLS) regression model was formulated for empirical analysis:


(5) $$\mathrm{health}=\alpha_{01}+\alpha_{11}\mathrm{access}01+\beta_{1}X+\varepsilon_{1}$$

(6) $$\mathrm{health}=\alpha_{02}+\alpha_{12}\mathrm{access}02+\beta_{2}X+\varepsilon_{2}$$

(7) $$\mathrm{welfare}=\alpha_{03}+\alpha_{13}\mathrm{access}01+\beta_{3}X+\varepsilon_{3}$$

(8) $$\mathrm{welfare}=\alpha_{04}+\alpha_{14}\mathrm{access}02+\beta_{4}X+\varepsilon_{4}$$

In the model, “health” signifies the self-rated health of the respondents, while “welfare” denotes the life satisfaction level of the respondents. “access01” indicates whether the respondents face unavailability of modern energy, and “access02” signifies the degree to which modern energy is inaccessible to respondents. “X” encompasses other control variables, such as respondents’ personal characteristics, property status, etc.

Equation (5) serves as a regression model to investigate the influence of energy unavailability on the health of middle-aged and older adults. Similarly, Equation (6) is formulated as a regression model to examine the impact of energy unavailability on their welfare.

Equation (7) is designed as a regression model to explore the effects of energy unavailability on the welfare of middle-aged and older adults. Likewise, Equation (8) is structured as a regression model to assess the impact of energy unavailability on their welfare.

### 4.2 Baseline regression analysis

**Figure 2:**
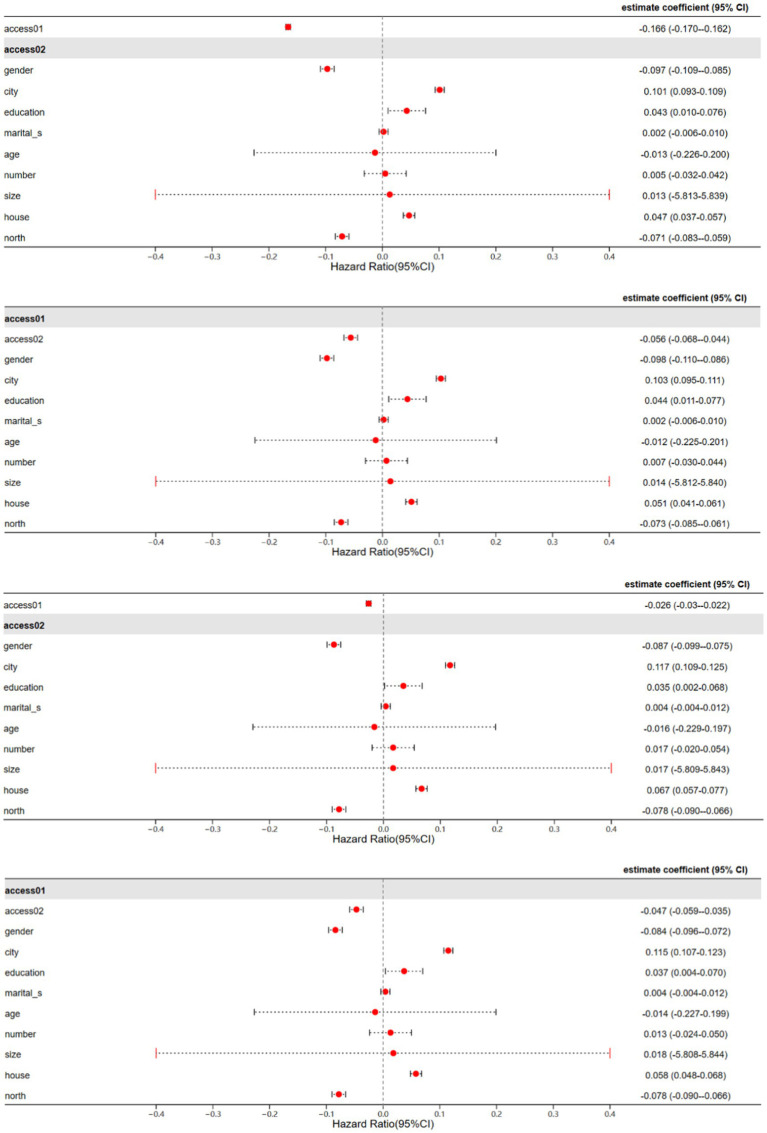
OLS regression results.

The baseline regression results obtained using the established econometric model and Stata software are presented in Table 3 (with province effects fixed in the regression) (Figure 2).

**Table 3:** OLS regression results.

	(1) Health	(2) health	(3) Welfare	(4) Welfare
Access01	−0.166*** (0.000)		−0.026*** (0.001)	
Access02		−0.056** (0.023)		−0.047*** (0.001)
Gender	−0.097** (0.015)	−0.098** (0.014)	−0.087** (0.024)	−0.084** (0.013)
City	0.101*** (0.009)	0.103*** (0.008)	0.117** (0.015)	0.115** (0.017)
Education	0.043*** (0.000)	0.044*** (0.000)	0.035*** (0.000)	0.037*** (0.000)
Marital_s	0.002 (0.157)	0.002 (0.150)	0.004 (0.174)	0.004 (0.187)
Age	−0.013*** (0.000)	−0.012*** (0.000)	−0.016*** (0.000)	−0.014*** (0.000)
Number	0.005** (0.027)	0.007** (0.029)	0.017** (0.035)	0.013** (0.035)
Size	0.013** (0.028)	0.014** (0.024)	0.017** (0.036)	0.018** (0.037)
House	0.047 (0.176)	0.051 (0.176)	0.067 (0.176)	0.058 (0.176)
North	−0.071** (0.046)	−0.073** (0.043)	−0.078** (0.049)	−0.078** (0.048)
Constant	2.114*** (0.000)	2.118*** (0.000)	2.115*** (0.000)	2.13*** (0.000)
Provincial dummy	yes	yes	yes	yes
N	6,748	6,748	6,748	6,748
$R^{2}$	0.16	0.16	0.16	0.16

***p < 0.01, **p < 0.05, *p < 0.1.

In Table 3, Models 1 and 2 explore how EP impacts the health of middle-aged and older adults. The findings reveal that the regression coefficients associated with access01 and access02 are both negative and statistically significant. This implies that the lack of access to energy and the extent of its unavailability have noteworthy detrimental effects on the health of middle-aged and older adults. Based on the MEPI index indicator system constructed in Section 3.1.1, this paper analyzes from the perspective of cooking fuel and concludes that the results stem from the widespread use of traditional solid fuels such as biomass and coal for cooking and heating among middle-aged and older adults experiencing EP. The direct combustion of these solid fuels generates a large amount of harmful gases, leading to indoor and outdoor air pollution, thereby significantly impacting the health of the middle-aged and older adults with respiratory system diseases.

Models 3 and 4 examine the impact of EP on the welfare of middle-aged and older adults. The regression results indicate significant negative effects of the unavailability and degree of unavailability of modern energy on the welfare of this demographic group. According to the capability approach theory proposed by Amartya Sen, an individual’s welfare depends on the functionings they are able to achieve and the set of capabilities to achieve those functionings. The unavailability of modern energy deprives middle-aged and older adults of various functions such as cooking, heating, and entertainment, which they are unable to achieve. Therefore, EP significantly undermines the welfare of middle-aged and older adults.

### 4.3 Endogeneity test

To ensure the consistency of Ordinary Least Squares (OLS) estimates, the model needs to satisfy the assumption of exogeneity of explanatory variables. However, in this study, endogeneity is highly likely to exist. On the one hand, the presence of omitted variables inevitably leads to the omission of certain explanatory variables. On the other hand, due to bidirectional causality, EP significantly negatively affects the health of middle-aged and older adults, but unhealthy individuals are also more likely to have lower incomes, thus being in a situation of EP.

To address endogeneity issues, this study introduces instrumental variables and utilizes them in Two-Stage Least Squares (TSLS) estimation. We use the community-level unavailability of energy as instrumental variables for Models 1 and 3, and the degree of unavailability of energy at the community level as instrumental variables for Models 2 and 4. Many scholars consider using the mean of the region to which the sample belongs as instrumental variables for individuals feasible, as the level of EP in the region is closely related to individual EP but unrelated to individual health and welfare levels.

To ensure the accuracy of the instrumental variable (IV) method, it is necessary to test for the presence of weak instruments. This is done by calculating the F-statistic for the first-stage regression. The results indicate that all F-statistics for the instrumental variables are greater than 10, indicating the absence of weak instruments.

Having passed the test, these instrumental variables are deemed effective, allowing us to employ them to test for endogeneity of the explanatory variables. Due to the presence of heteroscedasticity, we utilize the modified Durbin–Wu–Hausman test. The test results reveal that both the chi-square statistic and the F-statistic have *p*-values less than 0.05, indicating that Models 1–4 indeed have endogenous explanatory variables, and thus, the use of the IV method is justified.

**Figure 3:**
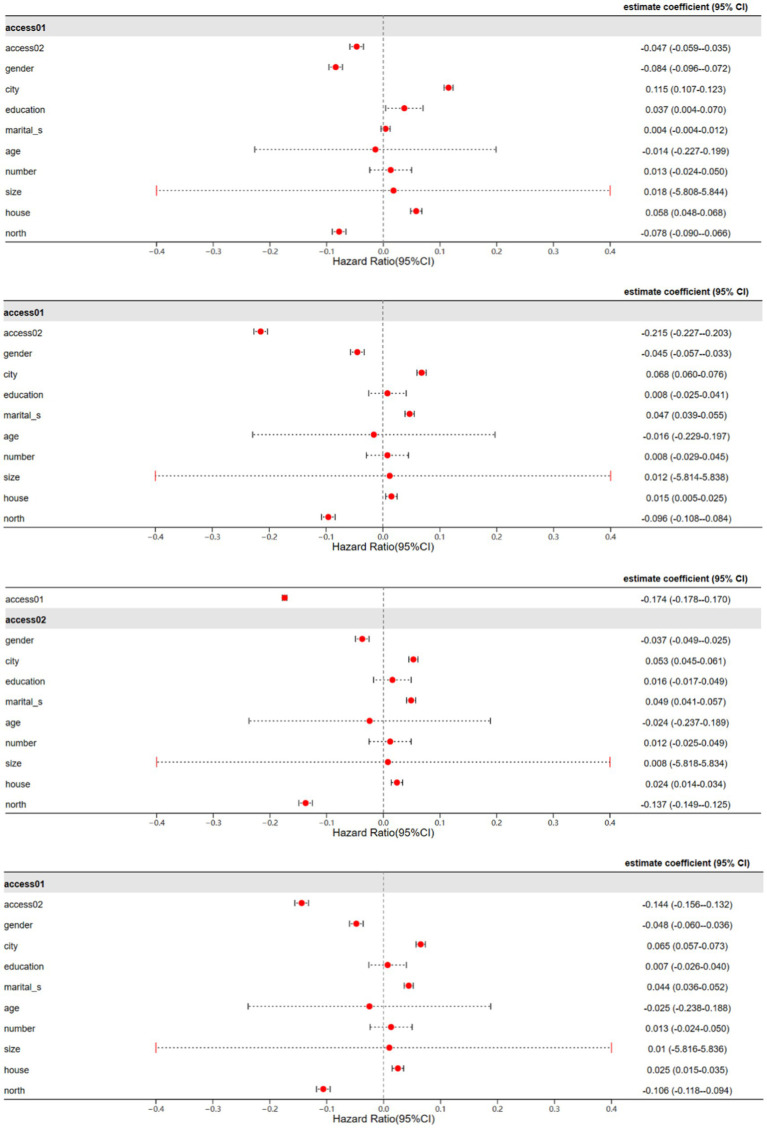
TSLS regression results.

Table 4 presents the results of the TSLS regressions after incorporating instrumental variables. The results indicate that there is no significant change in the regression results after employing the IV method. Both the unavailability and degree of unavailability of energy continue to exhibit significant negative effects on the health and welfare of middle-aged and older adults (Figure 3).

**Table 4:** TSLS regression results.

	(1) Health	(2) Health	(3) Welfare	(4) Welfare
Access01	−0.254*** (0.003)		−0.174*** (0.007)	
Access02		−0.215*** (0.004)		−0.144** (0.025)
Gender	−0.035** (0.026)	−0.045** (0.018)	−0.037** (0.024)	−0.048** (0.013)
City	0.059*** (0.005)	0.068*** (0.007)	0.053** (0.014)	0.065** (0.015)
Education	0.012*** (0.006)	0.008*** (0.009)	0.016*** (0.007)	0.007*** (0.009)
Marital_s	0.046 (0.137)	0.047 (0.126)	0.049 (0.136)	0.044 (0.138)
Age	−0.026*** (0.002)	−0.016*** (0.000)	−0.024*** (0.003)	−0.025*** (0.000)
Number	0.006** (0.045)	0.008** (0.035)	0.012** (0.046)	0.013** (0.037)
Size	0.015** (0.018)	0.012** (0.024)	0.008** (0.012)	0.01** (0.017)
House	0.013 (0.123)	0.015 (0.113)	0.024 (0.125)	0.025 (0.115)
North	−0.145** (0.036)	−0.096** (0.046)	−0.137** (0.034)	−0.106** (0.045)
Constant	1.334*** (0.000)	1.364*** (0.000)	1.436*** (0.000)	1.864*** (0.000)
Provincial dummy	yes	yes	yes	yes
N	6,748	6,748	6,748	6,748
Anderson LM	352.545***	264.643***	554.645***	456.337***
C-D-Wald	633.54*	345.533*	435.634*	453.433*

***p < 0.01, **p < 0.05, *p < 0.1.

### 4.4 Robustness test

To explore the robustness of the empirical results, we conduct a robustness analysis using alternative variables. As mentioned earlier, studying EP involves considering not only the unavailability but also the affordability of modern energy. Therefore, in this study, we replace the core explanatory variables with indicators of energy affordability and its degree. Specifically, we use afford01 and afford02 as the core explanatory variables and re-run the TSLS regressions. The regression results are presented in Table 5.

**Table 5:** TSLS regression results with replacement variables.

	(1) Health	(2) Health	(3) Welfare	(4) Welfare
Afford01	−0.353*** (0.004)		−0.164*** (0.003)	
Afford02		−0.253*** (0.004)		−0.154** (0.025)
Gender	−0.025** (0.025)	−0.054** (0.024)	−0.034** (0.032)	−0.043** (0.034)
City	0.054*** (0.003)	0.064*** (0.005)	0.075** (0.016)	0.065** (0.023)
Education	0.023*** (0.003)	0.013*** (0.005)	0.023*** (0.005)	0.014*** (0.005)
Marital_s	0.053 (0.234)	0.045 (0.245)	0.046 (0.134)	0.043 (0.143)
Age	−0.024*** (0.004)	−0.013*** (0.002)	−0.025*** (0.002)	−0.023*** (0.004)
Number	0.004** (0.034)	0.006** (0.035)	0.013** (0.034)	0.015** (0.025)
Size	0.023** (0.023)	0.025** (0.025)	0.034** (0.023)	0.023** (0.027)
House	0.023 (0.135)	0.022 (0.142)	0.025 (0.143)	0.026 (0.153)
North	−0.143** (0.046)	−0.093** (0.043)	−0.132** (0.045)	−0.142** (0.034)
Constant	2.355*** (0.000)	2.644*** (0.000)	2.453*** (0.000)	2.546*** (0.000)
Provincial dummy	Yes	Yes	Yes	Yes
N	6,748	6,748	6,748	6,748
Anderson LM	536.648***	365.646***	546.545***	455.553***
C-D-Wald	323.234*	534.332*	534.532*	425.432*

***p < 0.01, **p < 0.05, *p < 0.1.

**Figure 4:**
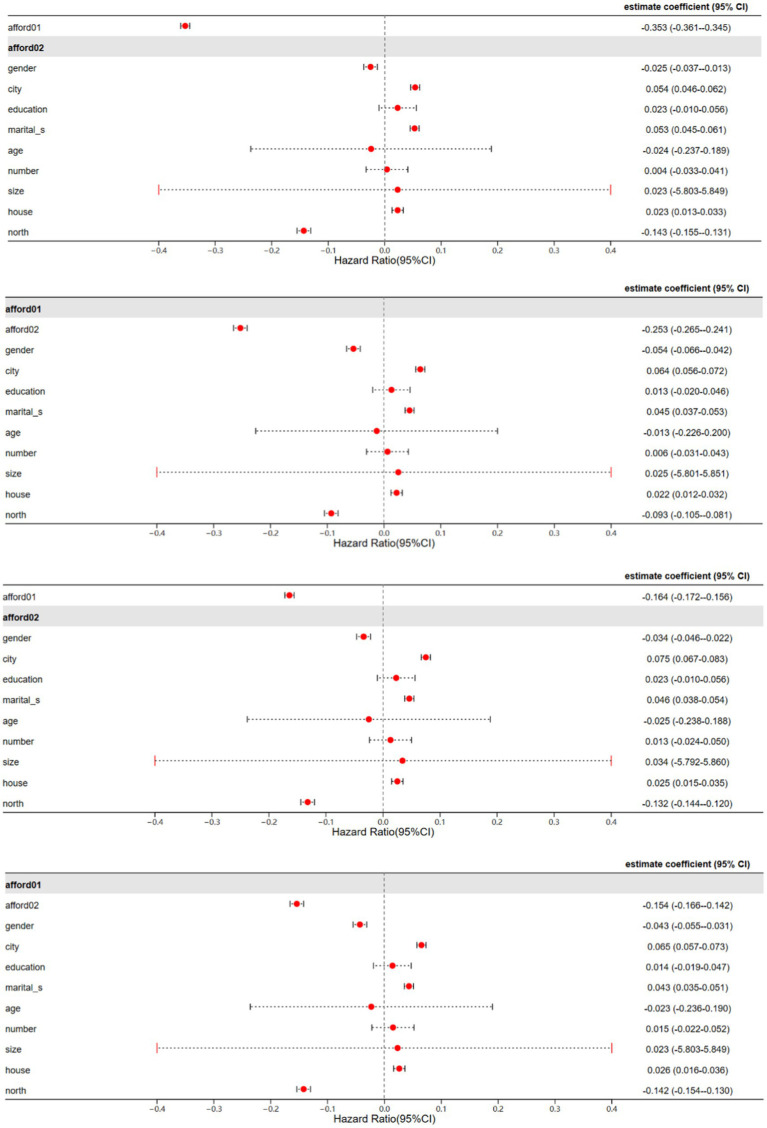
TSLS regression results with replacement variables.

From the regression results, it can be observed that after replacing the variables, the TSLS regressions show no significant changes. Using the 10% significance level, EP measured by affordability has a significant negative impact on the health and welfare of middle-aged and older adults. The deeper the level of energy unaffordability, the unhealthier the middle-aged and older adults, and the lower their subjective life satisfaction. Thus, it can be concluded that the findings of this study are robust (Figure 4).

## 5 Conclusion and policies

### 5.1 Conclusion

This study initially evaluated the prevalence of EP among China’s middle-aged and older adults in 2018, utilizing both the MEPI and the 10% indicator. The MEPI is a composite index that considers energy accessibility, while the 10% indicator assesses energy affordability. The findings reveal a widespread and severe issue of EP among this population segment. Based on MEPI calculations, 44% of the middle-aged and older adults are affected by EP, while the 10% indicator indicates an EP incidence rate of 43%. The 10% index shows a high sensitivity to energy prices, but the price of China’s energy market is relatively stable, and the price of solid fuels is relatively cheap. Therefore, poorer households can also afford it. This makes the 10% index more accurately reflect the EP status of middle-aged and older adults in China. Consequently, the results obtained from calculating the 10% indicator are similar to those of the MEPI indicator, both indicating the widespread impact of EP on the middle-aged and older adults in China. Furthermore, the study found that EP has a significant negative impact on the health and wellbeing of the middle-aged and older adults, and the deeper the EP, the more severe the impact on health and wellbeing. Even after conducting endogeneity and robustness analyses, the research results remained unchanged, further confirming the significance of this conclusion. EP exacerbates health issues among the middle-aged and older adults while also diminishing their overall wellbeing. This finding underscores the urgency of addressing the EP problem, particularly for the middle-aged and older adults.

During the formulation of econometric models, a comprehensive consideration of potential endogenous and exogenous factors was undertaken to ensure the precision and credibility of the findings. The utilization of these analytical approaches served to augment our comprehension of how EP impacts the health and overall welfare of middle-aged and older adults.

To summarize, this study underscores the extensive and severe nature of EP among China’s middle-aged and older adults, highlighting its detrimental consequences on their health and welfare. Such conclusions offer valuable insights for future initiatives aimed at ameliorating EP and enhancing the quality of life for this population segment.

### 5.2 Policies

EP, a critical yet often overlooked issue, disproportionately impacts the older adults, intertwining economic hardships with social equity and environmental sustainability. This multidimensional challenge requires a comprehensive approach to ensure that our most vulnerable populations are not left in the cold, quite literally. The following expanded recommendations aim to address EP with a focus on the older adults proposing actionable strategies that span from policy reforms to grassroots initiatives.

#### 5.2.1 Enhance energy services for older adults in rural areas

Due to limited sources of energy for older adults in rural areas, and the greater distance from urban areas where there is a wider range of household appliances in terms of quantity and type, the willingness of older adults in rural areas to use clean energy is often not strong enough. To alleviate this phenomenon, the government should strengthen the construction of basic energy service facilities in rural areas, establish a relatively complete energy service infrastructure and management system, and increase investment in energy for multidimensional energy-poverty older adults, making modern smart appliances more accessible in rural areas. Additionally, targeted and diversified energy service policies should be formulated to encourage diversified development of energy supply in rural areas, thereby increasing the willingness of older adults in rural areas to use clean energy. This will vigorously promote economic development in rural areas, effectively leverage the poverty-alleviating effects of overall economic development in rural areas, achieve sustainable development in energy consumption, and curb the further development of multidimensional EP in older adults in rural areas.

#### 5.2.2 Integrating household energy alternatives with targeted poverty alleviation

The intersection of illness-induced poverty poses one of the most daunting challenges to China’s poverty alleviation strategy. Rural residents who are relatively disadvantaged due to poverty, low education levels, and aging often face constraints on their ability to pay, leading them to rely more on traditional solid fuels. This reliance exposes them to higher health risks and increases the likelihood of falling into poverty traps due to energy structure issues. Therefore, within the framework of rural targeted poverty alleviation strategies, subsidies can be provided to impoverished and low-income rural households to use cleaner energy sources such as natural gas, fuel, and biogas. When setting poverty lines, consideration should be given to the cost of using clean energy. By integrating household energy alternatives with targeted poverty alleviation efforts, it’s possible to mitigate the health risks associated with traditional solid fuels, improve living standards, and break the cycle of poverty caused by energy-related challenges in rural areas.

#### 5.2.3 Enhancing monitoring mechanisms

The multidimensional EP levels among middle-aged and older adults in China exhibit significant regional disparities and universality. Therefore, it is recommended that the government and relevant departments address the distribution of multidimensional EP among middle-aged and older adults in urban and rural areas, as well as different regions. By doing so, they can establish targeted and graded monitoring mechanisms for EP, and promptly develop preventive strategies. For instance, based on the distribution of urban and rural areas and different regions, attention should be focused on vulnerable social groups in terms of EP levels, ensuring that no household is overlooked. Implementing grid-based and regional management strategies can effectively monitor and address EP. Considering factors such as transportation accessibility and population density, efforts should be made to strengthen energy infrastructure networks in villages with convenient transportation and high population density, promoting the extension of gas supply facilities to rural areas. In areas with limited transportation and scattered rural residences, emphasis should be placed on the development of biogas, solar energy, solar power generation, and wind power. By tailoring monitoring mechanisms and development strategies to the specific characteristics of different regions, the government can effectively address EP among middle-aged and older adults and promote sustainable development.

#### 5.2.4 Conduct energy education activities

In the context of EP, empowering the older adults through education is vital. Community centers and relevant organizations should provide practical activities related to energy efficiency, energy health, and sustainability. In promoting healthy environments, knowledge, and behaviors, there should be an emphasis on the environmental health hazards of solid fuels. This can inspire awareness and initiative among older adults in rural areas to seek alternative sources of household energy, while educating them about the benefits and methods of using clean energy. Enhancing the energy utilization knowledge of the older adults not only helps reduce EP and improve their health, but also promotes sustainable social development and raises awareness about energy.

## Data availability statement

The raw data supporting the conclusions of this article will be made available by the authors, without undue reservation.

## Author contributions

YWa: Writing – original draft, Writing – review & editing. YWu: Writing – original draft, Writing – review & editing. CW: Writing – original draft, Writing – review & editing. LL: Writing – original draft, Writing – review & editing. YL: Writing – original draft, Writing – review & editing. SW: Writing – original draft, Writing – review & editing. ZQ: Writing – original draft, Writing – review & editing.

## Footnotes

^1^The survey subjects of CHARLS are Chinese middle-aged and older adults and households over the age of 45, so this article will focus on studying this demographic.
